# Dr. Paul Fortunatus Mundé

**Published:** 1902-03

**Authors:** 


### OBITUARY.

Dr. Paul Fortunatus Mundé, of New York City, died February 7, 1902, from heart disease at his home, No. 20 West Forty-fifth Street, after an illness of two months.

Dr. Mundé was born in Dresden, Saxony, September 7, 1846. He was the son of Charles Mundé and Bertha von Hornemann-Mundé. The latter was the daughter of Baron von Hornemann, counselor of the King of Saxony. At the age of three years he was brought to this country by his parents, his father being a political refugee.

Paul F. Mundé was educated at home in his early years, then he was sent to the Public Latin School at Boston. In 1863 he entered Yale Medical Department. Leaving college before his course was completed he went to the civil war, serving as an acting medical cadet during six months of 1864. Afterward he entered Harvard Medical School, from which he was graduated in 1866. The same year he went to Europe and enlisted as a volunteer assistant surgeon on the Bavarian side in the war of 1866, receiving the cross of civil merit at its close.

When he was mustered out of the Bavarian army he went to Würzburg, becoming resident physician to the maternity hospital. It was here he came under the influence of Scanzoni, which probably shaped his destiny. He remained at Würzburg till 1870, when he joined the Bavarian army, in which he served as battalion surgeon, with the rank of first lieutenant, during the Franco-Prussian war. He received the iron cross from the Emperor for his heroism in removing patients from a burning hospital on the outskirts of Paris.

At the close of the war Dr. Mundé devoted himself to hospital work, receiving the degree of Master of Obstetrics from Vienna in December, 1871. He visited various hospitals of Europe in the next two years, including those at Heidelberg, Berlin, London, Edinburgh and Paris. He returned to this country and settled in New York in 1873, where he devoted himself to gynecology and consulting obstetrics.

In 1874 he became the editor of *American Journal of Obstetrics*, a position in which he continued until January, 1892. He was one of the founders of the American Gynecological Society, of which he was vice-president in 1898. He served as president of the New York Obstetrical Society from 1886 to 1888. He was elected vice-president of the British Gynecological Society in 1887. He was a member of the German Gynecological Society and a corresponding fellow of the obstetrical societies of Philadelphia, Leipsic and Edinburgh. He was a member of the Medical Society of the County of New York, and a fellow of the New York Academy of Medicine.

In 1897 Dr. Mundé received the degree of LL.D., from Dartsmouth, where he had been professor of gynecology since 1880. He belonged to the Union League, Riding and South Side Sportsman's Clubs.

Dr. Mundé was exceedingly fond of fishing. He made canoe trips of four days' duration into the St. John's Lake district, where the land-locked salmon fishing with a fly was famous, a number of years ago. He was the author of several authoritative books, and over one hundred journal articles, relating to obstetrics and gynecology. His treatise on minor surgical gynecology is known all over the world. Whenever and wherever gynecology is discussed the name of Mundé is mentioned, and his work as editor of the *American Journal of Obstetrics* referred to gave direction to American gynecology.

